# The Association of *Toxoplasma gondii* IgG and Cardiovascular Biomarkers

**DOI:** 10.3390/ijerph18094908

**Published:** 2021-05-05

**Authors:** Amani Babekir, Sayed Mostafa, Emmanuel Obeng-Gyasi

**Affiliations:** 1 Department of Built Environment, North Carolina A&T State University, Greensboro, NC 27411, USA; aebabekir@aggies.ncat.edu; 2 Environmental Health and Disease Laboratory, North Carolina A&T State University, Greensboro, NC 27411, USA; 3 Department of Mathematics and Statistics, North Carolina A&T State University, Greensboro, NC 27411, USA; sabdelmegeed@ncat.edu

**Keywords:** *Toxoplasma*, *T. gondii*, cardiovascular disease, biomarkers, cardiovascular health

## Abstract

*Background: Toxoplasma gondii* (*T. gondii*) is a protozoan parasite with high prevalence worldwide. More than 40 million individuals in the United States carry this parasite. *T. gondii* infection causes toxoplasmosis, which is the leading cause of death associated with foodborne diseases in the United States. *T. gondii* infects humans through different routes, and it is capable of invading a wide range of tissues in the human body following the infection. *Methods:* The main objective of this study was to investigate the prevalence of *T. gondii* among adults in the United States and its association with cardiovascular health using data from the National Health and Nutrition Examination Survey (NHANES 2009–2010). Considering the limitation of studies investigating the relationship between *T. gondii* and cardiovascular biomarkers, this study was focused on assessing the association of *T. gondii* to nine cardiovascular biomarkers. First, those biomarkers were investigated individually using several statistical tests and models. Second, we developed an overall cardiovascular biomarker index (OCBI) from eight critical biomarkers to better explain the *T. gondii* potential cumulative effect on the cardiovascular system. These analyses were adjusted for demographic, behavioral, and anthropometric factors. *Results:* *T. gondii* IgG antibody-positive participants had significantly higher systolic blood pressure (*p* = 0.0022), triglycerides (*p* = 0.0399), C-reactive protein (*p* = 0.0422), gamma glutamyl transferase (*p* = 0.0400), and fasting glucose (*p* = 0.0213) than the negative participants. In addition, the positive participants had significantly lower high-density lipoprotein cholesterol (*p* = 0.0431) than the negative participants. Adjusting for age, *T. gondii* positive had a significant negative association with high-density lipoprotein cholesterol (*p* = 0.0026) and a significant positive association with low-density lipoprotein cholesterol (*p* = 0.0179), triglycerides (*p* = 0.0154), and gamma glutamyl transferase (*p* = 0.0026). With the exception of the low-density lipoprotein, these associations remained statistically significant when adjusting for demographic, behavioral, and anthropometric factors. These results potentially indicate the role of *T. gondii* in driving cardiovascular-related biomarkers toward dysfunction. The analysis also revealed a significant difference in the OCBI among positive and negative participants (*p* = 0.0020), with the (cumulative) odds of positive participants having a higher level of OCBI being 0.71 times lower than the odds for negative participants (OR = 0.29). *Conclusions:* Positive *T. gondii* IgG antibody was significantly associated with adverse effects on cardiovascular-related biomarkers, including systolic blood pressure, low-density lipoprotein cholesterol, high-density lipoprotein cholesterol, triglycerides, and gamma glutamyl transferase. *T. gondii*-positive individuals were more likely to have a lower cardiovascular biomarkers index than the negative individuals. Finally, the prevalence of toxoplasmosis among U.S. adults was associated with demographic characteristics including age, ethnicity, country of birth, and occupation.

## 1. Introduction

### 1.1. Toxoplasmosis

*Toxoplasma gondii* (*T. gondii*) is a widespread protozoan parasite that infects one-third of the world population [1,2]. *T. gondii* causes toxoplasmosis, which is the leading cause of death related to foodborne diseases in the United States. More than 40 million individuals in the United States carry the parasite [3], with infection being more prevalent among older adults, foreign-born individuals, those with lower educational attainment, and individuals working in soil-related occupations [4,5]. Although most *T. gondii* infections are asymptomatic, it becomes a chronic infection when the parasite invades tissues and forms cysts [6].

The spread of toxoplasmosis in the United States is monitored by the Centers for Disease Control and Prevention (CDC) through the National Health and Nutrition Examination Survey (NHANES), which has revealed among other findings that race is a critical factor in determining who is exposed. Specifically, the prevalence of *T. gondii* is higher among non-Hispanic black Americans and Mexican Americans compared to non-Hispanic white Americans [7].

### 1.2. Transmission

The primary transmission of *T. gondii* to humans is through the consumption of food contaminated with *T. gondii* sporulated oocysts or by food contaminated with infected feline feces [8]. The life cycle of *T. gondii* includes three infectious forms of the parasite: the tachyzoites (which is rapid reproducing form), the bradyzoites (which is found in tissue cyst), and the sporozoites (which his found in oocysts). Wild and domestic cats are the main hosts, and they are capable of shedding millions of oocysts in their feces 3–14 days after the initial ingestion of any of the infectious forms [9]. When cats eat small animals or raw meat contaminated with tissue cysts, the stomach enzymes dissolve the cyst wall, and bradyzoites penetrate the small intestine wall to multiply and form oocysts. Then, the cells of the small intestine wall rupture and discharge these oocysts.

After excretion in the cat feces, the oocysts sporulate within 1 to 5 days and survive in normal environmental conditions for a long period of time and contaminate environmental media such as drinking water. They can also transmit to several animals consumed by cats and humans (intermediate hosts) [10,11,12].

Inside animals, the parasite invades tissues forming tissue cysts, where they survive for several years [13]. Humans become infected by eating undercooked meat that contains tissue cysts. They can also be infected through organ transplant or via congenital transmission [13,14].

### 1.3. Pathogenesis of Toxoplasmosis

After ingestion, digestive enzymes rupture the tissue cysts walls and release bradyzoites, which produce hundreds of tachyzoites. The tachyzoites are capable of infecting any nucleated cell in humans after entering the circulatory system and disseminating to organs and tissues; moreover, the tachyzoites cause cell death and inflammation [15,16]. *T. gondii* strains are subdivided into three major genotypes groups (strain types I, II, and III) with differences in virulence; however, the prevalence of these genotypes is characterized by global, regional, and local differences with strain type II being the most prevalent strain in the United States [17,18]. The virulent strains (e.g., strain types I) are associated with an increase in the frequency and severity of human toxoplasmosis; however, other factors such as human immune system status and genetic background may affect the susceptibility and severity of the infection [19].

### 1.4. T. gondii and Diseases

The human immune system usually prevents *T. gondii* from causing disease; however, pregnant women and immunocompromised individuals are more susceptible to sever infection [13,15]. When women become infected during pregnancy, the parasite may cross the placental barrier and infect the developing fetus, which may cause ocular disease, nervous system disease, or growth failure and abnormalities [8,15]. Human toxoplasmosis is associated with damage to various organs, including the liver, brain, and heart; moreover, immune deficiency can reactivate the latent parasite, causing more damages to the organs [16]. The global and systemic effects of *T. gondii* exposure are best seen in its effects on the nervous system. *Toxoplasma* tissue cysts in the brain have been found to alter the dopaminergic neuromodulatory system, increasing the risk of obsessive-compulsive disorder and schizophrenia [19]. This speaks to the potential effects of exposure throughout the life course.

Worldwide cardiovascular diseases are the leading cause of death, and in the United States, one individual dies every 36 s from cardiovascular disease [20,21]. Khademvatan et al. (2020) studied the association between *T. gondii* infection and cardiovascular diseases among a group of healthy individuals and cardiac patients in Iran. They tested the group for *T. gondii* antibody IgG by ELISA and found that the cardiovascular patients showed significantly higher (63.73%) anti-*Toxoplasma* IgG antibodies than the healthy volunteers (37.64%) (*p* < 0.001). This study indicated that heart failure might be exacerbated by toxoplasmosis. The parasite also invades various parts of the cardiovascular system causing myocarditis, cardiomyopathies, pericarditis, and pulmonary hypertension [22]. Previous studies indicated the association between *T. gondii* infection and elevated levels of biomarkers related to inflammation, dyslipidemia, cardiovascular events, vascular injury, and endothelial adhesion [23]. These studies focused on establishing associations between exposure to *T. gondii* and adverse health outcomes, and no clear causation was concluded. The current study primarily aims to further investigate this association, and it does not attempt to establish any causation conclusions.

### 1.5. Identification/Antibodies

The primary method to diagnose toxoplasmosis is serologic tests to detect specific antibody against *T. gondii* (IgG and IgM antibodies) [24]. The acute phase of toxoplasmosis induces high levels of IgM antibody and positive levels of IgG antibody usually within the first three weeks, but in the chronic phase, the level of IgM antibody decreases to normal values, and the level of IgG antibody increases [25]. Therefore, toxoplasmosis is diagnosed by the positive IgG antibody, which is an indication of acute or chronic infection with the IgM antibody used as a confirmatory test of the acute phase [26].

### 1.6. Cardiovascular-Related Clinical and Biomarkers

A biomarker is an attribute that is measured and evaluated as an indicator of a pathogenic process; therefore, several biomarkers are used as indicators to cardiovascular diseases [27]. The increase of triglycerides (TG) or total cholesterol (TC) is associated with cardiovascular disease; moreover, the increase of low-density lipoprotein cholesterol (LDL-C) is considered a major risk for cardiovascular dysfunction [28]. High systolic blood pressure (SBP) and diastolic blood pressure (DBP) cause blockages in the blood vessels and increase the heart workload, which could contribute to cardiovascular dysfunction [29]. C-reactive protein (CRP) is an acute-phase protein that is stimulated by pro-inflammatory cytokines; and it is a biomarker of systemic inflammations and cardiovascular disease [30]. Therefore, the presence of CRP in the serum is a good predictor of cardiovascular complications. [31]. Gamma glutamyl transferase (GGT) is usually found on the external surface of cell membranes and its increase is an indication of the oxidative stress, which is an imbalance between the creation and elimination of reactive oxygen species (ROS) in the cells [32]. Thus, GGT can be used as a biomarker for adverse cardiovascular events [33]. In addition, the American Heart Association recommends the fasting glucose (FG) biomarker as an indicator for cardiovascular health.

### 1.7. Purpose

There are limited studies investigating the association between toxoplasmosis and cardiovascular disease risk in the United States population, and very few studies have explored this association using the cardiovascular-related biomarkers, which are important tools to evaluate risk levels and speculate on mechanisms of action. Thus, the purpose of this study is to comprehensively explore the association between *Toxoplasma gondii* IgG antibody and various cardiovascular biomarkers in a representative sample of U.S. adults to better understand the health complications of toxoplasmosis. This is critical, since it will help improve our understanding of lesser-known contributors to cardiovascular dysfunction and will contribute to identifying all the factors that have made cardiovascular diseases the leading cause of mortality in the United States and worldwide.

## 2. Materials and Methods

### 2.1. Hypothesis

This study hypothesized that *T. gondii* infection is associated with adverse levels of cardiovascular biomarkers. The first objective was to examine the association between *T. gondii* and various demographic, behavioral, and anthropometric variables such as gender, age, ethnicity, country of birth, occupation, alcohol consumption, smoking, and BMI. Subsequently, we examined the association between *T. gondii* exposure and cardiovascular health biomarkers such as SBP, DBP, HDL, LDL, TG, TC, CRP, GGT, and FG among the adult population of the United States.

### 2.2. Study Population

This study’s sample was extracted from the NHANES 2009–2010 sample to focus on participants who were 20 years or older and tested for *T. gondii* in that survey round. NHANES is a multistage stratified survey designed to provide a detailed examination of the health and nutritional status of a nationally representative sample of non-institutionalized individuals in the United States. This study used de-identified secondary data; hence, the study did not require IRB approval. The protocol for NHANES 2009–2010 (Continuation of Protocol #2005-06) was approved by the National Center for Health Statistics Research Ethics Review Board (ERB); and all participants gave informed consent. Sampling was done in four stages: sampling counties, sampling segments, sampling households, and sampling persons. The U.S. counties were divided into 15 groups based on their characteristics, and one county was then selected from each group.

### 2.3. Demographics

The demographic information was collected through NHANES computer-assisted personal interview (CAPI) software program. The collected information included gender, age, race/ethnicity, country of birth, and occupation.

### 2.4. Clinical Variables (Biomarkers)

#### 2.4.1. Blood Pressure

Blood pressure measurements, including SBP and DBP, were taken during the examination. Three consecutive blood pressure readings were taken after sitting for 5 min and obtaining the maximum inflation level.

#### 2.4.2. Oxidative Stress and Systemic Inflammation

Blood specimens were tested for CRP in University of Washington, Seattle, WA. The method used to quantify CRP was latex-enhanced nephelometry. Samples were sent to Collaborative Laboratory Services for analysis to test GGT, and the enzymatic rate method was used to determine the GGT activity in serum or plasma.

#### 2.4.3. Cholesterol and Triglycerides

LDL cholesterol and TG were measured using a Roche Modular P chemistry analyzer (University of Minnesota, Minneapolis, MN, USA). For cholesterol, the HDL measuring method included the addition of magnesium/dextran sulfate solution to the sample to form water-soluble complexes (University of Minnesota, Minneapolis, MN, USA). HDL-cholesterol esters were converted to HDL-cholesterol. Total cholesterol was calculated by adding LDL cholesterol, HDL cholesterol, and 20% of TG.

#### 2.4.4. Glucose (FG)

Diabetes was tested by measures of fasting plasma glucose in the morning. Blood specimens were collected and shipped to Fairview Medical Center Laboratory at the University of Minnesota for analysis.

#### 2.4.5. Exposure Variable: *T. gondii*

The antibodies of *T. gondii* were analyzed with specific *Toxoplasma* IgG enzyme immunoassay kit (Bio-Rad, Redmond, VA, USA), which had high sensitivity and specificity. Results were reported as IU/mL and coded as positive (≥33 IU/mL) or negative (<27 IU/mL). Samples with equivocal results (≥27 IU/mL and <33 IU/mL) were repeated twice and confirmed as negative.

### 2.5. Covariates

Additional variables were considered in the data analysis, including taking prescription medications for high cholesterol/hypertension, physical activity, BMI, gender, age, alcohol, and smoking. The physical activity information such as daily activities was collected through a questionnaire that was adapted from the Global Physical Activity Questionnaire.

### 2.6. Overall Cardiovascular Biomarkers Index (OCBI)

An overall cardiovascular biomarkers index (OCBI) was created following the methodology utilized by the American Heart Association [34]. Eight biomarkers were used in the index, including biomarkers recommended by the American Heart Association [34]. Each biomarker was given an index value (0 or 1) based on the cutoff value recommended by the American Heart Association and clinical practices, assigning the value 0 to the unacceptable values of the biomarkers. The overall biomarker index was the sum of the individual biomarker index values (Table 1).

### 2.7. Statistical Analysis

Each research hypothesis was evaluated using the related variables to examine the association with *T. gondii*. The statistical analysis was conducted using R (version 4.0.2; R Foundation for Statistical Computing, Vienna, Austria). The analysis accounted for the survey weights and the sampling design of NHANES through the use of the Survey package in R, which is specifically designed to analyze complex survey data. Descriptive statistics were calculated to summarize the variables included in the analysis. Rho–Scott chi-square bivariate analyses, design-based *t*-tests, Tukey multiple comparisons of means, and logistic regression were used to evaluate the associations between *T. gondii* IgG and the cardiovascular biomarkers. Our analysis adjusted for demographic, behavioral, and anthropometric covariates; and a *p*-value < 0.05 was considered significant in all our analyses.

## 3. Results

Table 2 provides descriptive summaries (survey-weighted percentages and means) of the variables included in the analysis. The total number of sample participants was 5324 (48.1% male and 51.9% female). The portion of positive *T. gondii* among the sample participants was 15.2%. The average age of participants was 47.2 years. The sample consisted of 8.7% Mexican American, 5.1% other Hispanic, 69.5% non-Hispanic white, 10.4% non-Hispanic black, and 6.4% other race. Considering country of birth, 81.5% of participants were born in the U.S. The weighted means of the cardiovascular biomarkers are also detailed in Table 2.

**Figure 1:**
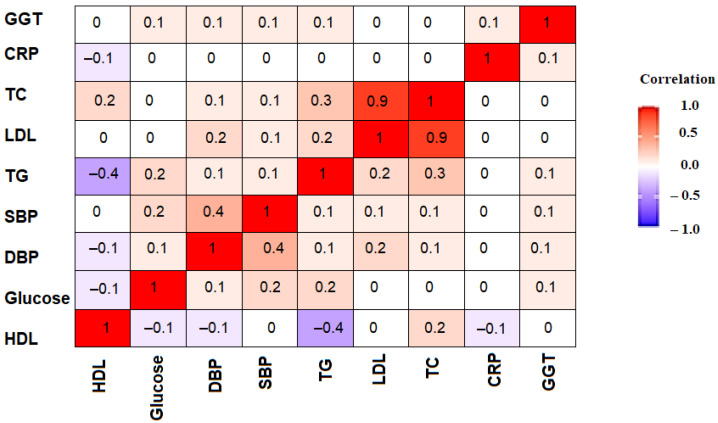
Cardiovascular biomarkers correlation matrix.

A correlation matrix was created to explore the correlation between the cardiovascular biomarkers (Figure 1). Overall, the biomarkers appear to have low correlations except for the strong correlation between TG and LDL cholesterol (Pearson correlation coefficient *ρ* = 0.9).

The design-based *t*-test and the Rho–Scott chi-square test were used to examine the association between the *T. gondii* IgG antibody status (positive/negative) and the demographic factors. The results displayed in Table 3 showed that there was a statistically significant association between the *T. gondii* status and each of age, race/ethnicity, country of birth, and occupation (*p* < 0.001). The mean age of *T. gondii* positive participants was significantly higher than the mean age of negative participants. Other Hispanic had the biggest proportion of *T. gondii*-positive individuals among the ethnic groups. The proportion of *T. gondii*-positive individuals was the highest among individuals born in other Spanish-speaking countries who worked in the agriculture, forestry, fishing, mining, or construction industries. The association between the *T. gondii* status and sex was not statistically significant at the 5% significance level.

To further investigate the variability in *T. gondii* IgG antibody among the different levels of race/ethnicity and country of birth, we conducted multiple comparisons of means (with Tukey adjustment) of the *T. gondii* level (Table 4). Other Hispanic had a higher level of *T. gondii* IgG antibody when compared with Mexican Americans, non-Hispanic whites, and non-Hispanic blacks (*p* < 0.0001), while Mexican Americans had a higher level of *T. gondii* IgG antibody than non-Hispanic whites (*p* < 0.0001). On the other hand, non-Hispanic blacks had a higher level of *T. gondii* IgG antibody than non-Hispanic whites (*p* < 0.0001). Considering country of birth, individuals born in Mexico and other Hispanic or non-Hispanic countries had higher levels of *T. gondii* positive than those who were born in the U.S. (*p* < 0.0001).

The design-based *t*-test was used to explore the impact of the *T. gondii* IgG antibody status (positive/negative) on each of the cardiovascular biomarkers (Table 5). *T. gondii* IgG antibody positive participants had significantly higher SBP (*p* = 0.0022), TG (*p* = 0.0399), CRP (*p* = 0.0422), GGT (*p* = 0.0400), and FG (*p* = 0.0213) than the negative participants. In addition, the positive participants had significantly lower HDL (*p* = 0.0431) than the negative participants.

Table 6 summarizes the results of several linear regression models that were fit to further assess the association between each of the cardiovascular biomarkers and the *T. gondii* IgG antibody status while adjusting for demographic variables and/or covariates. Three models were fit for each biomarker. The first model was adjusted for age, and the second model was adjusted for age and other demographic factors (gender, race, and country of birth). The third model was adjusted for age, several covariates, including anthropometric (BMI), and behavioral factors (alcohol use and smoking). The results in Table 6 show that *T. gondii* positive has significant negative correlation with HDL (*p* = 0.0026) and significant positive correlation with LDL (*p* = 0.0179), TG (*p* = 0.0154), and GGT (*p* = 0.0026). This correlation was apparent in all three models except for the LDL model adjusted for the demographic factors (*p* = 0.1071). The models for DBP, TC, CRP, and FG did not show significant correlation with the *T. gondii* IgG antibody status.

### Overall Cardiovascular Biomarkers Index (OCBI)

To examine the association between *T. gondii* and cardiovascular biomarkers, we constructed the OCBI index. This index was created based on eight out of the nine biomarkers investigated in this study including SBP, DBP, HDL, TG, TC, CRP, GGT, and FG. The biomarker LDL was excluded from the index due to its strong correlation with TG, which was revealed in the correlation analysis (Figure 1). The index point range was from 0 to 8 (see Table 7). The highest weighted sample proportion was on point 6, while points 0, 1 and 2 had very a minor number of participants and population proportions. There was a difference in the OCBI level among positive and negative participants with the positive participants being more likely to have low OCBI.

To overcome the limitation of the created OCBI due to the small sample sizes at the low levels of the index (i.e., 0, 1, and 2), two subindexes were created (OCBI-Subindex1 and OCBI-Subindex2). OCBI-Subindex1 (Table 8) was created by aggregating the cases in levels 0, 1, 2, and 3 into one class (≤3). The analysis on OCBI-Subindex1 showed that there was a significant difference in the OCBI levels among positive and negative participants (*p* = 0.0209), again with the positive participants being more likely to have low OCBI.

Ordinal logistic regression models were used to model the OCBI-Subindex1 on the *T. gondii* IgG antibody status (positive/negative), while adjusting for the demographic variables and covariates (see Table 9 and Table 10). Positive *T. gondii* IgG antibody was inversely associated with change in OCBI-Subindex1 (OR = 0.29, 95% CI = 0.12–0.68, *p* = 0.0468) when adjusting for demographic factors, with positive subjects having 71% lower odds of reporting an increase in OCBI-Subindex1 compared to negative subjects (i.e., positive subjects were more likely to have lower levels of OCBI-Subindex1 than negative subjects). When adjusting for covariates, positive *T. gondii* IgG antibody was inversely associated with change in OCBI-Subindex1 (OR = 0.24, 95% CI = 0.09–0.69, *p* = 0.0287). In both models, a significant interaction between *T. gondii* and age was observed (*p* = 0.0279 and *p* = 0.0194 for the two models, respectively). However, adjusting the model for taking prescription medications for cholesterol and hypertension did not show a significant association between the OCBI-Subindex1 and the *T. gondii* IgG antibody status (Table 11). It should be noted that the sample size for the model adjusted for taking prescription medications was much smaller than the sample size used for the other models due to lack of data.

The OCBI-Subindex2 (Table 12) was created by using the median of the OCBI (point 6) as a cutoff to create two levels of the subindex (≤6 was assigned point 0 and >6 was assigned point 1). OCBI-Subindex1 showed that there was a significant difference in the OCBI points among positive and negative participants (*p* = 0.0379), there was a smaller proportion of ideal OCBI (point 8) among positive participants when compared to the negative group (0.26 vs. 0.33).

In addition, a binary logistic regression model of OCBI-Subindex2 on *T. gondii* IgG antibody (positive/negative) after adjusting for the demographic factors and covariates (Table 13) showed the inverse association of *T. gondii* with OCBI-Subindex2 (OR = 0.31, 95% CI = 0.12–0.83) and age interaction in this association (*p* = 0.0315).

## 4. Discussion

### 4.1. Overview and Implications of Results

This study’s findings indicate that *T. gondii* exposure among U.S. adults is associated with demographics characteristics, including gender, age, ethnicity, country of birth, and occupation. These findings confirm the current literature, which found that Latinos of any race have higher odds of testing positive for *T. gondii* than other ethnicities [38]. Our study revealed that other Hispanics are more likely to have higher *T. gondii* IgG antibody levels than Mexican Americans, and individuals born in other countries (outside of the U.S.) have the highest level of *T. gondii* IgG antibody. Furthermore, the study participants who worked in agriculture, forestry, fishing, mining, and construction fields have a higher prevalence of *T. gondii* than the other fields. This high prevalence could be due to the contamination with *Toxoplasma* oocysts in these settings [10].

A previous study suggested a correlation between *Toxoplasma* infection and cardiovascular diseases [39]. However, this study was not in the USA population. Our study used a comprehensive list of biomarkers and investigated this correlation using a nationally representative sample from the NHANES database. A recent study showed an association between *T. gondii* IgG antibody and elevated biomarkers of chronic inflammation and vascular injury, including CRP [23], but they limited the study to a sample from North Carolina.

Our study confirmed the significant difference of CRP among *Toxoplasma* negative and positive groups and investigated a broader range of biomarkers linked to cardiovascular system health. This analysis showed that positive *T. gondii* IgG antibody was positively associated with elevated SBP, TG, GGT, and FG, and negatively associated with HDL.

After adjusting this association for age, demographic factors, and other covariates (behavioral and anthropometric factors), our analysis confirmed the positive association between *T. gondii* infection and an increase in the level of LDL, TG, and GGT in addition to a significant negative association with the level of HDL.

After investigating each biomarker individually, an overall cardiovascular biomarkers index (OCBI) was created following the methodology of the American Heart Association for cardiovascular health (CVH). Additional biomarkers were added to this OCBI including HDL, GGT, and CRP. The biomarker HDL was added to the index because of its association with cardiovascular health and the negative association with *Toxoplasma*, which was revealed in the initial analysis. GGT and CRP were added because previous studies linked these biomarkers to cardiovascular health [32,33].

The predictive models created with OCBI and *Toxoplasma* showed the association between them. This association was still evident when adjusting the models for the demographic, behavioral, and anthropometric factors. Even though *Toxoplasma* potentially contributes to cardiovascular dysfunction, factors such as dietary habits, fitness and physical activity, genetic conditions, and other underlying diseases significantly affect cardiovascular health and must remain a priority in mitigating cardiovascular disease risk. That said, potentially lesser contributing factors such as *Toxoplasma* must be considered as mechanistic processes resulting from exposure seems to potentially contribute to cardiovascular disease.

The generated indexes and models indicate that *Toxoplasma*-positive subjects have lower odds of reporting healthier cardiovascular index than the negative subjects. Our findings revealed the interaction between age and the adverse effect of *Toxoplasma* in cardiovascular health. This finding supported *Toxoplasma* and age interaction, which was reported in previous studies [40].

**Figure 2:**
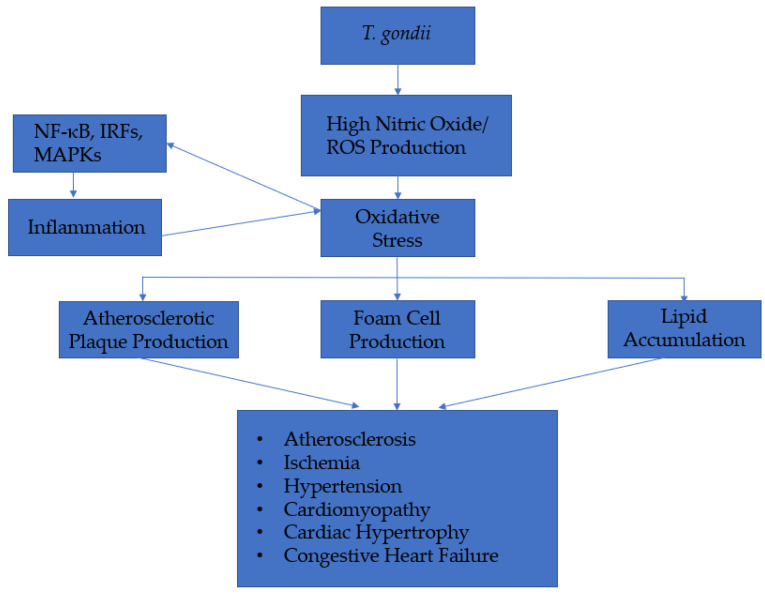
Mechanism of action of *T. gondii* on cardiovascular-related dysfunction.

The mechanism by which *T. gondii* infection causes cardiovascular dysfunction is complex, but critical trends are observed, giving credence to the findings of this study (Figure 2). The infection of *T. gondii* is linked to an increase in oxidative stress and inflammation, which has an adverse cardiovascular outcome. The inflammatory response is mediated through nuclear factor κB (NF-κB), IFN regulatory factors (IRFs), and mitogen-activated protein kinases (MAPKs). These three pathways play critical roles in the induction of interleukin (IL)12, IL1β, interferons (IFN) type I and type II, and tumor necrosis factor α (TNFα) [41,42].

Prior studies have indicated that *Toxoplasma* infection increases the production of nitric oxide (NO) and ROS, leading to an increase in vasoconstriction and oxidative stress in the tissues [43]. The oxidative stress increases atherosclerotic plaque production, foam cell formation, lipid accumulation, and inflammation [32]. The adverse cardiovascular outcomes of the oxidative stress includes LDL oxidation, endothelial injury, vascular modeling, and platelet activation [32]. It must be noted that there exist marked differences in the inflammatory response against different strain types of *Toxoplasma* and the immune response against *Toxoplasma* varies depending on the genetic background and immune status of the host. Nevertheless, Figure 2 offers critical insight into how exposure to *Toxoplasma* may contribute to cardiovascular dysfunction.

### 4.2. Public Health Implications

Heart disease and stroke are among the leading causes of death in the USA. Lifestyle factors are well-known contributors to adverse cardiovascular outcomes, but environmental and microbial factors are less well studied. Our results add to the literature on the cardiovascular disease exposome [44] and suggest that *T. gondii* and potentially other parasites must be considered as a factor when assessing the risk of cardiovascular disease. The effects of *T. gondii*, combined with other cardiovascular disease comorbidities, could have an immense effect on specific populations.

### 4.3. Limitations

This was a cross-sectional study using data collected at one specific point in time. Since temporality of association is a strong criterion for causality, this study cannot prove causality, but it helps to generate a causal hypothesis of the role of *T. gondii* on the cardiovascular health. A future longitudinal study will be ideal in confirming the associations revealed in this study. Although the sample size used in this study is representative to the U.S. population, it is a small fraction of the U.S. adult population, and caution should be taken when interpreting these results. Finally, one is rarely exposed to *T. gondii* alone; other factors in combination with *T. gondii* may have synergistic effects, which may alter cardiovascular health.

## 5. Conclusions

In summary, positive *T. gondii* IgG antibody is significantly associated with adverse clinical cardiovascular biomarkers including HDL, LDL, TG, and GGT. Additionally, positive *T. gondii* IgG antibody is significantly associated with the adverse overall cardiovascular biomarkers index created from clinical cardiovascular-related biomarkers. Finally, *Toxoplasma* prevalence in the U.S. adult population is associated with demographic variables such as age, ethnicity, country of birth, and occupation.

## Figures and Tables

**Table 1:** Overall cardiovascular biomarkers index (OCBI).

Cardiovascular Biomarker	Biomarker Value Assigned Index Value (1)	Biomarker Value Assigned Index Value (0)	Reference
Diastolic blood pressure (DBP)	≤80	>80	[34]
Systolic blood pressure (SBP)	≤120	>120	[34]
High-density lipoprotein (HDL)	≥50 mg/dL	<50 mg/dL	[35]
Triglycerides (TG)	≤199 mg/dL	>199 mg/dL	[34]
C-reactive protein (CRP)	≤1 mg/dL	>1 mg/dL	[36]
Gamma glutamyl transferase (GGT)	≤40 U/L	>40 U/L	[37]
Total cholesterol (TC)	<200 mg/dL	>200 mg/dL	[34]
Glucose (FG)	<100 mg/dL	>100 mg/dL	[34]
Overall biomarkers index	Sum of the index values		[34]

**Table 2:** Sampled data characteristics, NHANES 2009–2010, *n* = 5324.

Variables	*n*	Weighted Percentage/Mean (SE)
*Toxoplasma gondii* IgG antibody		
<33 IU/mL (negative)	4301	84.8
≥33 IU/mL (positive)	1023	15.2
Gender		
Male	2581	48.1
Female	2743	51.9
Age	5324	47.2 (0.49)
Race/ethnicity		
Mexican American	993	8.7
Other Hispanic	552	5.1
Non-Hispanic White	2627	69.5
Non-Hispanic Black	876	10.4
Other race	276	6.4
Country of birth		
Born in US	3847	81.5
Born in Mexico	628	5.4
Born in other Spanish-speaking country	406	3.8
Born in other non-Spanish-speaking country	441	9.2
Occupation		
Agriculture, forestry, fishing, mining, construction	163	10.2
Others	1277	89.8
Alcohol use		
Yes	799	16.1
No	3382	83.9
Cigarette use		
Yes	2489	45.1
No	2835	54.9
Activity (Vigorous)		
Yes	969	19.9
No	4355	80.1
Blood pressure medicine		
Yes	1639	86.8
No	222	13.2
Cholesterol medicine		
Yes	983	84.8
No	171	15.0
BMI	5272	28.83 (0.13)
Cardiovascular biomarker		
Blood pressure		
Systolic	4753	120.63 (0.46)
Diastolic	4753	69.23 (0.62)
High-density lipoprotein	5311	53.00 (0.40)
Low-density lipoprotein	2525	116.01 (1.07)
Total cholesterol	5311	196.25 (0.98)
Triglycerides	5304	151.50 (2.16)
C-reactive protein	5324	0.38 (0.02)
Gamma glutamyl transaminase	5304	1.71 (0.02)
Glucose	2573	104.20 (0.85)

**Table 3:** Associations between *T. gondii* (positive/negative) and demographic variables.

Variable	*T. gondii* Negative		*T. gondii* Positive		*p*-Value *
	*n*	Weighted Percentage or Mean (SE)	*n*	Weighted Percentage or Mean (SE)	
Age	1855	46.6 (0.59)	454	53.2 (0.87)	<0.001
Gender					
Male	2030	83.2	551	16.8	0.0532
Female	2271	86.3	472	13.7	
Race/ethnicity					
Mexican American	800	81.1	193	18.9	<0.001
Other Hispanic	361	68.1	191	31.9	
Non-Hispanic White	2208	86.9	419	13.1	
Non-Hispanic Black	705	82.1	171	17.9	
Other race	227	85.2	49	14.8	
Country of Birth					
Born US	3308	88.0	539	12.0	<0.001
Born in Mexico	461	72.7	167	27.3	
Born in other Spanish country	228	60.5	178	39.5	
Born in other non-Spanish country	302	74.1	139	25.9	
Occupation					
Agriculture, forestry, fishing, mining, construction	117	75.7	46	24.3	<0.001
Others	1039	84.4	238	15.6	

* The *p*-value from the design-based *t*-test for numerical variables and from the Rho–Scott chi-square test for categorical variables.

**Table 4:** Tukey multiple comparisons of means analysis of *T. gondii* IgG antibody among different race/ethnicity and country of birth.

Variable	Compared Levels		Mean Difference	*p*-Value
Race/ethnicity	Other Hispanic	Mexican American	18.35	<0.0001
	Non-Hispanic White	Mexican American	−8.50	<0.0001
	Non-Hispanic Black	Mexican American	−0.57	0.9993
	Other race	Mexican American	−7.36	0.2168
	Non-Hispanic White	Other Hispanic	−26.52	<0.0001
	Non-Hispanic Black	Other Hispanic	−18.91	<0.0001
	Other race	Other Hispanic	−25.70	<0.0001
	Non-Hispanic Black	Non-Hispanic White	7.94	<0.0001
	Other race	Non-Hispanic White	1.14	0.9967
	Other race	Non-Hispanic Black	−6.79	0.3077
Country of birth	Born in Mexico	Born in US	20.35	<0.0001
	Born in other Spanish country	Born in US	38.95	<0.0001
	Born in other non-Spanish country	Born in US	17.73	<0.0001
	Born in other Spanish country	Born in Mexico	18.60	<0.0001
	Born in other non-Spanish country	Born in Mexico	−2.62	0.9188
	Born in other non-Spanish country	Born in other Spanish-speaking country	−21.21	<0.0001

**Table 5:** Associations between *T. gondii* (positive/negative) and cardiovascular biomarkers.

Variable	*T. gondii* Negative		*T. gondii* Positive		*p*-Value
	*n*	Weighted Mean (SE)	*n*	Weighted Mean (SE)	
Systolic blood pressure	3855	120.19 (0.48)	898	123.14 (0.84)	0.0022
Diastolic blood pressure	3855	69.35 (0.61)	898	68.52 (0.78)	0.0877
High-density lipoprotein	4290	53.31 (0.42)	1021	51.26 (0.87)	0.0431
Low-density lipoprotein	2026	116.26 (1.31)	499	114.71 (1.23)	0.4542
Total cholesterol	4290	196.35 (1.22)	1021	195.67 (0.96)	0.7206
Triglycerides	4285	150.07 (2.19)	1019	159.56 (4.42)	0.0399
C-reactive protein	4301	0.37 (0.02)	1023	0.42 (0.03)	0.0422
Gamma glutamyl transaminase	4285	1.69 (0.02)	1019	1.80 (0.05)	0.0400
Glucose	2061	103.67 (0.90)	512	107.01 (1.28)	0.0213

**Table 6:** Linear regression models to explain the associations between *T. gondii* (positive/negative) and cardiovascular biomarkers (adjusted for demographic or behavioral and anthropometric factors).

Response	Model Adjusted Factors *	*T. gondii* Positive	
		Coefficient (SE)	*p*-Value
Systolic blood pressure	Age	4.36 (2.08)	0.0564
	Age, gender, race, country of birth	2.44 (2.31)	0.3501
	Age, alcohol use, cigarette use, activity, BMI	2.37 (2.24)	0.3212
Diastolic blood pressure	Age	3.40 (3.55)	0.3561
	Age, gender, race, country of birth	2.47 (3.50)	0.5201
	Age, alcohol use, cigarette use, activity, BMI	2.89 (3.46)	0.4295
High-density lipoprotein	Age	−8.08 (2.17)	0.0026
	Age, gender, race, country of birth	−5.16 (1.75)	0.0421
	Age, alcohol use, cigarette use, activity, BMI	−8.33 (2.71)	0.0153
Low-density lipoprotein	Age	16.02 (5.91)	0.0179
	Age, gender, race, country of birth	12.00 (5.79)	0.1071
	Age, alcohol use, cigarette use, activity, BMI	20.23 (7.73)	0.0307
Triglycerides	Age	58.54 (21.01)	0.0154
	Age, gender, race, country of birth	51.81 (17.73)	0.0432
	Age, alcohol use, cigarette use, activity, BMI	64.74 (25.64)	0.0356
Total cholesterol	Age	14.69 (8.77)	0.1180
	Age, gender, race, country of birth	12.71 (8.55)	0.2114
	Age, alcohol use, cigarette use, activity, BMI	19.97 (10.71)	0.0993
C-reactive protein	Age	0.03 (0.16)	0.8190
	Age, gender, race, country of birth	−0.05 (0.15)	0.7479
	Age, alcohol use, cigarette use, activity, BMI	−0.15 (0.19)	0.4629
Gamma glutamyl transferase	Age	21.02 (5.65)	0.0026
	Age, gender, race, country of birth	17.59 (5.67)	0.0361
	Age, alcohol use, cigarette use, activity, BMI	24.40 (5.42)	0.0020
Glucose	Age	3.43 (4.12)	0.4210
	Age, gender, race, country of birth	0.11 (3.54)	0.9775
	Age, alcohol use, cigarette use, activity, BMI	−0.34 (3.30)	0.9196

* The behavioral factors (alcohol use and cigarette use) had a smaller number of data points than the other factors; therefore, it was evaluated in a separate model to minimize loss of data.

**Table 7:** Overall index of cardiovascular biomarkers (OCBI).

	OCBI Index									
Index Point	0	1	2	3	4	5	6	7	8	Total
Sample proportion	0.00	0.01	0.02	0.07	0.12	0.19	0.25	0.22	0.14	
*Toxoplasma* IgG antibody										
Negative (*n*)	0	14	39	118	234	407	438	370	235	1855
Positive (*n*)	1	2	12	44	64	105	106	87	33	454
Positive proportion	0.00	0.00	0.03	0.10	0.14	0.23	0.23	0.19	0.07	
Negative proportion	0.00	0.01	0.02	0.06	0.13	0.22	0.24	0.20	0.13	

**Table 8:** Overall index of cardiovascular biomarkers (OCBI-Subindex1).

OCBI-Subindex1						
Index Point	≤3	4	5	6	7	8
Population proportion	0.09	0.12	0.19	0.25	0.22	0.14
*Toxoplasma* IgG antibody						
Negative (*n*)	171	234	407	438	370	235
Positive (*n*)	59	64	105	106	87	33
Positive proportion	0.13	0.14	0.23	0.23	0.19	0.07
*p*-Value = 0.0209						

**Table 9:** Ordinal logistic regression model for the OCBI-Subindex1 on *T. gondii* IgG antibody (positive/negative) adjusted for the demographic factors.

	OCBI-Subindex1			
	Odds Ratio	OR 95% Confidence Interval		*p*-Value
*Toxoplasma* IgG antibody (positive)	0.29	0.12	0.68	0.0468
Age (years)	0.96	0.96	0.97	0.0007
Gender				
Male (ref)				
Female	2.79	2.30	3.39	0.0005
Race/ethnicity				
Mexican American (ref)				
Other Hispanic	1.28	0.70	2.33	0.4650
Non-Hispanic White	1.90	1.10	3.29	0.0839
Non-Hispanic Black	1.18	0.67	2.07	0.6039
Other race	1.65	0.72	3.77	0.3034
Country of birth				
Born in 50 US States (ref)				
Born in Mexico	1.24	0.81	1.90	0.3705
Born in other Spanish-speaking country	1.18	0.69	2.00	0.5835
Born in other non-Spanish-speaking country	1.32	0.94	1.86	0.1857
IgG antibody (positive): Age (years)	1.02	1.01	1.04	0.0279
Sample size = 2309				

**Table 10:** Ordinal logistic regression model for the OCBI-Subindex1 on *T. gondii* IgG antibody (positive/negative) adjusted for the covariates.

	OCBI-Subindex1			
	Odds Ratio	OR 95% Confidence Interval		*p*-Value
*Toxoplasma* IgG antibody (positive)	0.24	0.09	0.69	0.0287
Age (years)	0.97	0.96	0.98	0.0002
Alcohol use				
Yes (ref)				
No	1.70	1.35	2.12	0.0017
Cigarette use				
Yes (ref)				
No	1.02	0.89	1.18	0.7435
Activity				
Yes (ref)				
No	1.37	1.11	1.71	0.0206
BMI	0.89	0.87	0.92	0.0001
IgG antibody (positive): Age (years)	1.03	1.01	1.05	0.0194
Sample size = 1842				

**Table 11:** Ordinal logistic regression model for the OCBI-Subindex1 on *T. gondii* IgG antibody (positive/negative) adjusted for medications taken.

	OCBI-Subindex1			
	Odds Ratio	OR 95% Confidence Interval		*p*-Value
*Toxoplasma* IgG antibody (positive)	0.72	0.03	15.24	0.8378
Age (years)	1.01	0.98	1.04	0.6682
Taking cholesterol medicine				
Yes (ref)				
No	0.27	0.14	0.54	0.0037
Taking high blood pressure medicine				
Yes (ref)				
No	3.53	0.53	23.74	0.2231
IgG antibody (positive): Age (years)	1.01	0.97	1.06	0.5856
Sample size = 357				

**Table 12:** Overall index of cardiovascular biomarkers (OCBI-Subindex2).

	OCBI-Subindex2	
Index Point	0	1
Population proportion	0.65	0.35
*Toxoplasma* IgG antibody		
Negative (*n*)	1250	605
Positive (*n*)	334	120
Positive proportion	0.74	0.26
Negative proportion	0.67	0.33
*p*-Value = 0.0379		

**Table 13:** Binary logistic regression model for the OCBI-Subindex2 on *T. gondii* IgG antibody (positive/negative) adjusted for demographic factors and the covariates (after model selection).

	OCBI-Subindex2			
	Odds Ratio	OR 95% Confidence Interval		*p*-Value
Intercept	25.87	8.03	83.32	0.0055
*Toxoplasma* IgG antibody (positive)	0.31	0.12	0.83	0.0797
Age (years)	0.95	0.94	0.96	0.0008
BMI	0.89	0.86	0.93	0.005
Gender				
Male (ref)				
Female	2.84	2.10	3.86	0.0025
Race/ethnicity				
Mexican American (ref)				
Other Hispanic	1.05	0.74	1.48	0.8024
Non-Hispanic White	1.45	0.98	2.16	0.1378
Non-Hispanic Black	0.94	0.63	1.38	0.7554
Other race	0.88	0.41	1.91	0.7704
Alcohol use				
Yes (ref)	1.48	0.96	2.27	0.1471
No	0.00	0.00	0.00	0.0018
Activity				
Yes (ref)				
No	1.38	0.97	1.96	0.1469
IgG antibody (positive): Age (years)	1.03	1.01	1.05	0.0315
Sample size = 1842				
R2 = 0.30				

## Data Availability

The NHANES dataset is publicly available online, accessible at cdc.gov/nchs/nhanes/index.htm (accessed on 3 September 2020).
